# Supplementary material for: Integrated Transcriptional and Metabolomic Analysis of Factors Influencing Root Tuber Enlargement during Early Sweet Potato Development
Source: Genes (Basel). 2024 Oct 14;15(10):1319. doi: 10.3390/genes15101319 (PMC11507034; doi:10.3390/genes15101319)
Supplement: Supplementary file 1 [file genes-15-01319-s001.zip › Figure S1.pdf]

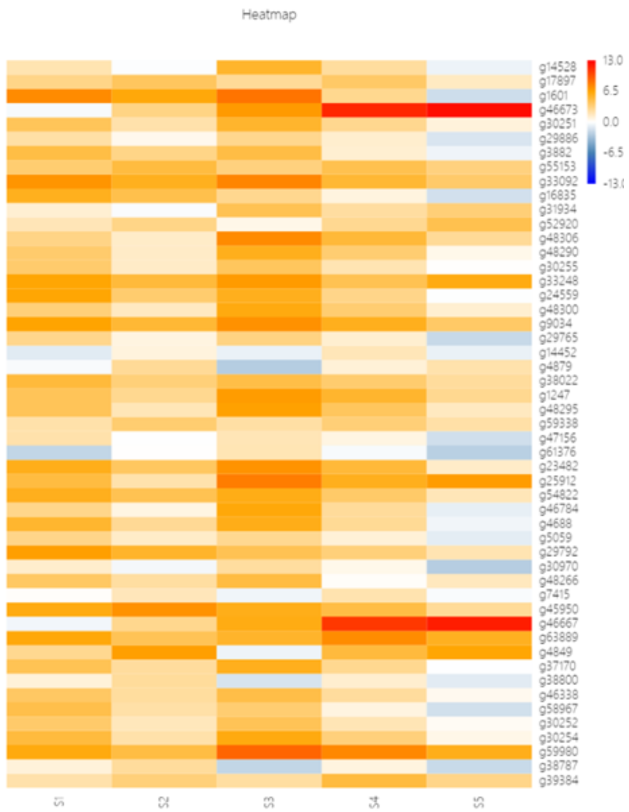


**Figure S1.** Heat map of expression of differentially expressed genes shared by the four comparison groups (S1 vs S2, S2 vs S3, S3 vs S4, and S4 vs S5) at different periods.
