# Supplementary material for: Integrated Transcriptional and Metabolomic Analysis of Factors Influencing Root Tuber Enlargement during Early Sweet Potato Development
Source: Genes (Basel). 2024 Oct 14;15(10):1319. doi: 10.3390/genes15101319 (PMC11507034; doi:10.3390/genes15101319)
Supplement: Supplementary file 1 [file genes-15-01319-s001.zip › Figure S3.pdf]

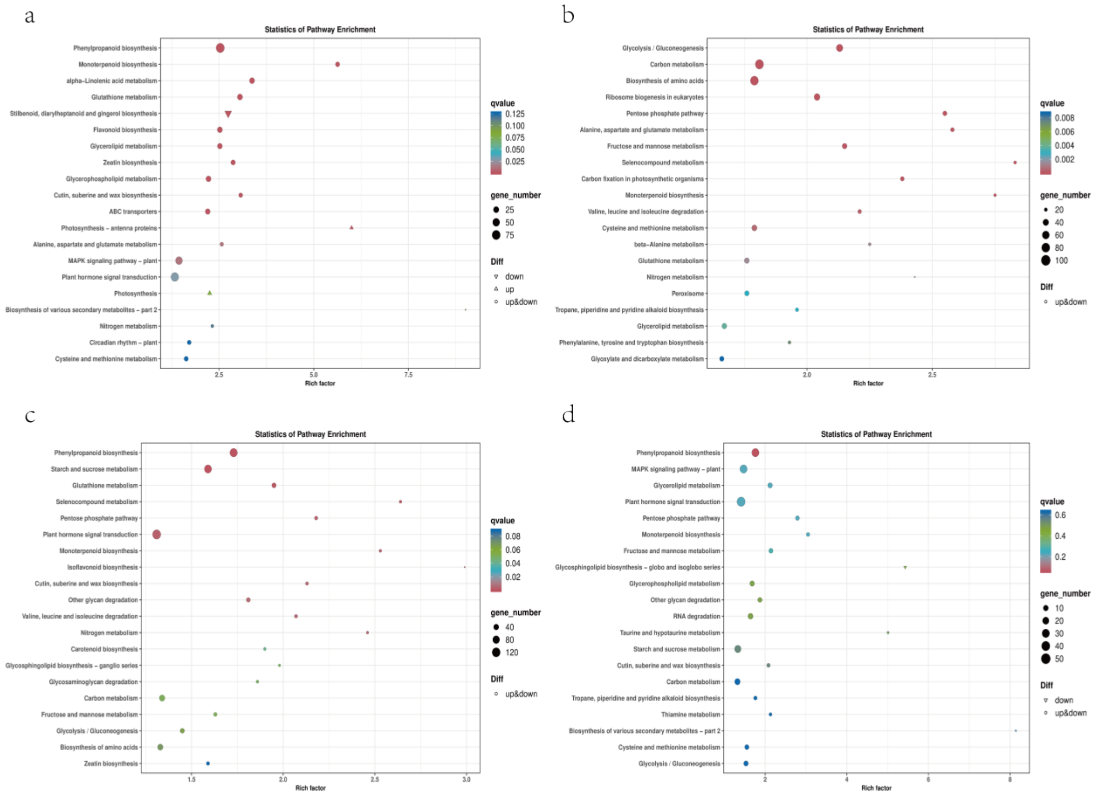

Figure S3. KEGG enrichment analysis of differentially expressed genes in four comparison groups. (**a**) S1 vs S2, (**b**)S2 vs S3, (**c**)S3 vs S4, (**d**)S4 vs S5
