# Supplementary material for: Integrated Transcriptional and Metabolomic Analysis of Factors Influencing Root Tuber Enlargement during Early Sweet Potato Development
Source: Genes (Basel). 2024 Oct 14;15(10):1319. doi: 10.3390/genes15101319 (PMC11507034; doi:10.3390/genes15101319)
Supplement: Supplementary file 1 [file genes-15-01319-s001.zip › Figure S4.pdf]

a

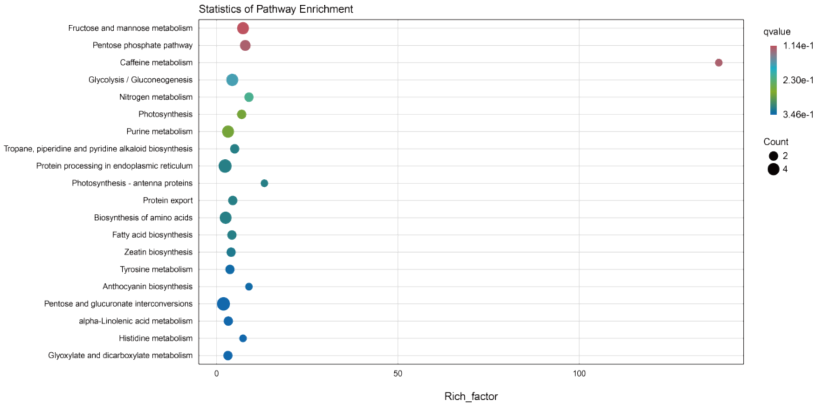

b

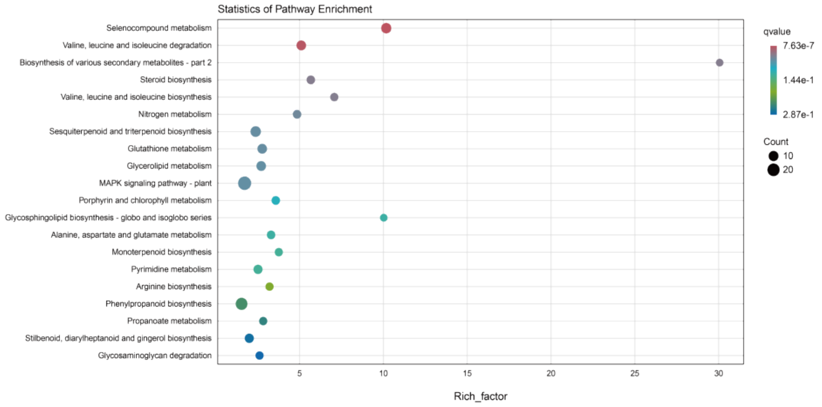

c

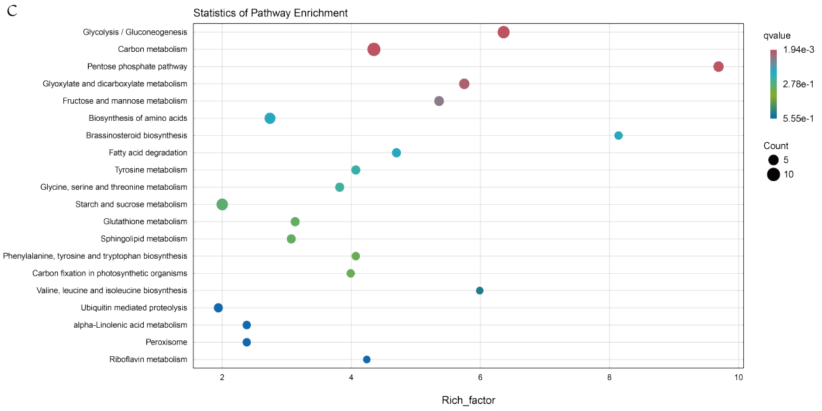

Figure S4. KEGG enrichment map of differentially expressed genes screened based on WGCNA. (**a**) black module, (**b**) brown module, (**c**) red module.
