# Supplementary material for: Integrated Transcriptional and Metabolomic Analysis of Factors Influencing Root Tuber Enlargement during Early Sweet Potato Development
Source: Genes (Basel). 2024 Oct 14;15(10):1319. doi: 10.3390/genes15101319 (PMC11507034; doi:10.3390/genes15101319)
Supplement: Supplementary file 1 [file genes-15-01319-s001.zip › Table S1.pdf]

Table S1. Transcriptome data of sweet potato root samples at different developmental stages.

Sample	ReadSum pair-end Reads	BaseSum	Q20(%)	Q30(%)	GC(%)
**S1-1**	21685289	6490294865	98.48	96.05	46.17
**S1-2**	19974111	5978904690	98.35	95.78	46.25
**S1-3**	20992982	6285100956	98.27	95.57	46.23
**S2-1**	20213157	6049570655	98.06	94.92	46.18
**S2-2**	19549548	5848667783	98.4	95.9	46.31
**S2-3**	19452871	5822566607	98.26	95.55	45.83
**S3-1**	20598034	6166171923	98.23	95.51	45.79
**S3-2**	20749492	6211884068	98.28	95.68	46.13
**S3-3**	20641143	6177242233	98.23	95.61	45.96
**S4-1**	20150870	6031661501	98.33	95.73	46.15
**S4-2**	20198697	6044017855	98.34	95.73	46.19
**S4-3**	19945402	5966060627	98.62	96.29	47.01
**S5-1**	19921202	5962323444	98.34	95.74	46.7
**S5-2**	23208982	6944658023	98.28	95.56	46.92
**S5-3**	22261992	6663324984	98.32	95.74	46.57
