# Supplementary material for: Integrated Transcriptional and Metabolomic Analysis of Factors Influencing Root Tuber Enlargement during Early Sweet Potato Development
Source: Genes (Basel). 2024 Oct 14;15(10):1319. doi: 10.3390/genes15101319 (PMC11507034; doi:10.3390/genes15101319)
Supplement: Supplementary file 1 [file genes-15-01319-s001.zip › Table S2.pdf]

Table S2. Comparison of transcriptome data with reference genomes.

Sample	Clean Reads	Mapped Reads	Uniq Mapped Reads	Multiple Map Reads
**S1-1**	43,370,578	35,569,575 (82.01%)	33,857,129 (78.06%)	1,712,446 (3.95%)
**S1-2**	39,948,222	33,000,379 (82.61%)	31,399,191 (78.60%)	1,601,188 (4.01%)
**S1-3**	41,985,964	34,559,887 (82.31%)	32,902,899 (78.37%)	1,656,988 (3.95%)
**S2-1**	40,426,314	32,920,789 (81.43%)	31,434,946 (77.76%)	1,485,843 (3.68%)
**S2-2**	39,099,096	30,982,892 (79.24%)	29,582,992 (75.66%)	1,399,900 (3.58%)
**S2-3**	38,905,742	31,186,042 (80.16%)	29,776,334 (76.53%)	1,409,708 (3.62%)
**S3-1**	41,196,068	33,732,246 (81.88%)	32,081,943 (77.88%)	1,650,303 (4.01%)
**S3-2**	41,498,984	33,954,455 (81.82%)	32,426,230 (78.14%)	1,528,225 (3.68%)
**S3-3**	41,282,286	33,801,685 (81.88%)	32,270,764 (78.17%)	1,530,921 (3.71%)
**S4-1**	40,301,740	33,158,675 (82.28%)	31,245,026 (77.53%)	1,913,649 (4.75%)
**S4-2**	40,397,394	32,892,645 (81.42%)	30,978,228 (76.68%)	1,914,417 (4.74%)
**S4-3**	39,890,804	32,043,561 (80.33%)	30,078,226 (75.40%)	1,965,335 (4.93%)
**S5-1**	39,842,404	32,857,066 (82.47%)	30,570,060 (76.73%)	2,287,006 (5.74%)
**S5-2**	46,417,964	37,869,638 (81.58%)	35,022,696 (75.45%)	2,846,942 (6.13%)
**S5-3**	44,523,984	36,914,441 (82.91%)	34,310,192 (77.06%)	2,604,249 (5.85%)
