# Supplementary material for: Integrated Transcriptional and Metabolomic Analysis of Factors Influencing Root Tuber Enlargement during Early Sweet Potato Development
Source: Genes (Basel). 2024 Oct 14;15(10):1319. doi: 10.3390/genes15101319 (PMC11507034; doi:10.3390/genes15101319)
Supplement: Supplementary file 1 [file genes-15-01319-s001.zip › Table S3.pdf]

Table S3. Differential expression genes shared by the four comparison groups (S1 vs S2, S3 vs S4, and S4 vs S5).

gene_name	nr_symbol	NR_annotation
**g1247**	LOC116021136	uncharacterized protein LOC116021136 [Ipomoea triloba]
**g14452**	LOC109177308	PREDICTED: uncharacterized protein LOC109177308 [Ipomoea nil]
**g14528**	LOC116002687	isoflavone reductase homolog isoform X3 [Ipomoea triloba]
**g1601**	LOC116020778	21 kDa protein-like [Ipomoea triloba]
**g16835**	LOC115999792	nudix hydrolase 18, mitochondrial-like [Ipomoea triloba]
**g17897**	--	--
**g23482**	LOC116006016	respiratory burst oxidase homolog protein B [Ipomoea triloba]
**g24559**	LOC116007152	cationic peroxidase 1-like [Ipomoea triloba]
**g25912**	LOC116013809	1-aminocyclopropane-1-carboxylate oxidase 1 [Ipomoea triloba]
**g29765**	LOC116012116	GDSL esterase/lipase At5g37690-like [Ipomoea triloba]
**g29792**	LOC116013949	8-hydroxygeraniol dehydrogenase-like [Ipomoea triloba]
**g29886**	LOC116013947	transcription factor MYB53-like [Ipomoea triloba]
**g30251**	LOC115996242	kunitz trypsin inhibitor 5-like, partial [Ipomoea triloba]
**g30252**	LOC115996363	kunitz trypsin inhibitor 5-like [Ipomoea triloba]
**g30254**	LOC115996242	kunitz trypsin inhibitor 5-like, partial [Ipomoea triloba]
**g30255**	LOC115996074	kunitz trypsin inhibitor 5-like [Ipomoea triloba]
**g30970**	LOC115996237	laccase-7-like [Ipomoea triloba]
**g31934**	LOC115995618	heavy metal-associated isoprenylated plant protein 7-like [Ipomoea triloba]
**g33092**	LOC115997346	kunitz trypsin inhibitor 5-like [Ipomoea triloba]
**g33248**	LOC115997358	bidirectional sugar transporter N3-like [Ipomoea triloba]
**g37170**	LOC116032229	berberine bridge enzyme-like 8 [Ipomoea triloba]
**g38022**	LOC116031905	uncharacterized protein LOC116031905 [Ipomoea triloba]
**g38787**	LOC116027810	1-aminocyclopropane-1-carboxylate synthase 11-like [Ipomoea triloba]
**g38800**	LOC116027810	1-aminocyclopropane-1-carboxylate synthase 11-like [Ipomoea triloba]
**g3882**	LOC109154996	PREDICTED: arogenate dehydrogenase 1, chloroplastic-like isoform X2 [Ipomoea nil]
**g39384**	LOC116027259	7-ethoxycoumarin O-deethylase-like [Ipomoea triloba]
**g45950**	LOC116018961	WRKY transcription factor 71-like [Ipomoea triloba]
**g46338**	LOC116020012	uncharacterized protein LOC116020012 [Ipomoea triloba]
**g46667**	--	sporamin [Ipomoea batatas]
**g46673**	--	sporamin B [Ipomoea batatas]
**g46784**	LOC115996016	ankyrin repeat-containing protein ITN1-like isoform X1 [Ipomoea triloba]
**g4688**	--	short-chain alcohol dehydrogenase [Ipomoea trifida]
**g47156**	LOC116024890	cinnamoyl-CoA reductase-like SNL6 [Ipomoea triloba]
**g48266**	LOC116025678	transcription factor MYB59-like isoform X1 [Ipomoea triloba]
**g48290**	LOC115997616	kunitz trypsin inhibitor 5-like [Ipomoea triloba]
**g48295**	LOC115997616	kunitz trypsin inhibitor 5-like [Ipomoea triloba]
**g48300**	LOC115997616	kunitz trypsin inhibitor 5-like [Ipomoea triloba]
**g48306**	LOC115996069	kunitz trypsin inhibitor 5-like [Ipomoea triloba]

**g4849**	LOC116016311	18.2 kDa class I heat shock protein-like [Ipomoea triloba]
**g4879**	LOC116015915	17.4 kDa class I heat shock protein-like [Ipomoea triloba]
**g5059**	LOC109163061	PREDICTED: GDSL esterase/lipase At2g30220-like [Ipomoea nil]
**g52920**	--	--
**g54822**	LOC116005475	probable glucan 1,3-beta-glucosidase A isoform X1 [Ipomoea triloba]
**g55153**	LOC116010306	subtilisin-like protease SBT1.5 [Ipomoea triloba]
**g58967**	LOC116029038	berberine bridge enzyme-like 26 [Ipomoea triloba]
**g59338**	LOC116028746	ankyrin repeat domain-containing protein 13C [Ipomoea triloba]
**g59980**	LOC116021783	uncharacterized endoplasmic reticulum membrane protein C16E8.02-like [Ipomoea triloba]
**g61376**	LOC116022595	transcription factor MYB58-like [Ipomoea triloba]
**g63889**	LOC116022984	aquaporin PIP2-4-like [Ipomoea triloba]
**g7415**	LOC116016688	WAT1-related protein At3g30340-like [Ipomoea triloba]
**g9034**	LOC109181978	PREDICTED: probable glutathione S-transferase [Ipomoea nil]
