# Supplementary material for: Integrated Transcriptional and Metabolomic Analysis of Factors Influencing Root Tuber Enlargement during Early Sweet Potato Development
Source: Genes (Basel). 2024 Oct 14;15(10):1319. doi: 10.3390/genes15101319 (PMC11507034; doi:10.3390/genes15101319)
Supplement: Supplementary file 1 [file genes-15-01319-s001.zip › Table S4.pdf]

Table S4. Differentially expressed genes screened by transcriptome WGCNA.

#ID	nr_symbol	NR_annotation
**g39013**	LOC116027933	purine permease 3-like [Ipomoea triloba]
**g778**	LOC116020705	germin-like protein subfamily 1 member 13 [Ipomoea triloba]
**g9270**	LOC116015445	glyoxylate/hydroxypyruvate reductase HPR3-like [Ipomoea triloba]
**g54313**	LOC116009900	ribokinase [Ipomoea triloba]
**g795**	LOC116020820	putative germin-like protein 2-1 [Ipomoea triloba]
**g42961**	LOC116024931	ferredoxin-dependent glutamate synthase, chloroplastic [Ipomoea triloba]
**g30713**	LOC116020705	germin-like protein subfamily 1 member 13 [Ipomoea triloba]
**g58562**	LOC116029295	triacylglycerol lipase SDP1-like [Ipomoea triloba]
**g5021**	LOC116017568	6-phosphofructo-2-kinase/fructose-2,6-bisphosphatase-like [Ipomoea triloba]
**g34770**	LOC116033646	peroxisomal (S)-2-hydroxy-acid oxidase-like [Ipomoea triloba]
**g5225**	LOC116016488	alcohol dehydrogenase 1-like [Ipomoea triloba]
**g26551**	LOC116013826	cycloartenol synthase-like isoform X4 [Ipomoea triloba]
**g39707**	LOC116027733	glucose-6-phosphate isomerase, cytosolic [Ipomoea triloba]
**g23327**	LOC116007206	ribulose bisphosphate carboxylase small chain clone 512-like [Ipomoea triloba]
**g42361**	LOC109169903	PREDICTED: alcohol dehydrogenase 3 [Ipomoea nil]
**g59572**	LOC116028770	squalene monooxygenase SE1-like [Ipomoea triloba]
**g58854**	LOC116028750	pyruvate decarboxylase 1-like [Ipomoea triloba]
**g30498**	LOC115997067	cycloartenol-C-24-methyltransferase 1-like [Ipomoea triloba]
**g21456**	LOC116006347	ATP-dependent 6-phosphofructokinase 6-like [Ipomoea triloba]
**g20547**	LOC115998516	L-idonate 5-dehydrogenase-like [Ipomoea triloba]
**g50604**	LOC109152449	PREDICTED: 2-oxoisovalerate dehydrogenase subunit beta 1, mitochondrial [Ipomoea nil]
**g57649**	LOC116029295	triacylglycerol lipase SDP1-like [Ipomoea triloba]
**g46531**	LOC116027165	pyrophosphate--fructose 6-phosphate 1-phosphotransferase subunit beta-like [Ipomoea triloba]
**g14527**	LOC116002687	isoflavone reductase homolog isoform X3 [Ipomoea triloba]
**g34133**	LOC116032367	methylcrotonoyl-CoA carboxylase beta chain, mitochondrial [Ipomoea triloba]
**g11789**	LOC116004381	2-isopropylmalate synthase A-like [Ipomoea triloba]
**g55289**	LOC116010460	malonate--CoA ligase-like [Ipomoea triloba]
**g25771**	LOC116014434	pyruvate kinase 1, cytosolic-like [Ipomoea triloba]
**g42970**	LOC116024931	ferredoxin-dependent glutamate synthase, chloroplastic [Ipomoea triloba]
**g58124**	LOC116028569	pyruvate kinase 1, cytosolic-like [Ipomoea triloba]
**g6458**	LOC116017142	lipoamide acyltransferase component of branched-chain alpha-keto acid dehydrogenase complex, mitochondrial [Ipomoea triloba]
**g30712**	LOC116020705	germin-like protein subfamily 1 member 13 [Ipomoea triloba]
**g29041**	LOC109208879	PREDICTED: uncharacterized protein LOC109208879 isoform X1 [Nicotiana attenuata]
**g793**	LOC109189050	PREDICTED: germin-like protein subfamily 1 member 13 [Ipomoea nil]
**g17158**	LOC115999636	branched-chain amino acid aminotransferase 2, chloroplastic-like [Ipomoea triloba]
**g42371**	LOC109169903	PREDICTED: alcohol dehydrogenase 3 [Ipomoea nil]

**g41978**	LOC116022427	branched-chain-amino-acid aminotransferase 2, chloroplastic-like isoform X1 [Ipomoea triloba]
**g782**	LOC116020705	germin-like protein subfamily 1 member 13 [Ipomoea triloba]
**g11788**	LOC116004381	2-isopropylmalate synthase A-like [Ipomoea triloba]
**g23312**	LOC116007206	ribulose bisphosphate carboxylase small chain clone 512-like [Ipomoea triloba]
**g799**	LOC116029278	germin-like protein subfamily 1 member 14 [Ipomoea triloba]
**g47071**	LOC116025277	pyrophosphate--fructose 6-phosphate 1-phosphotransferase subunit alpha-like [Ipomoea triloba]
**g798**	LOC116029278	germin-like protein subfamily 1 member 14 [Ipomoea triloba]
**g42425**	LOC116028332	2-oxoisovalerate dehydrogenase subunit alpha 1, mitochondrial-like isoform X1 [Ipomoea triloba]
**g16700**	LOC109191713	PREDICTED: xylose isomerase [Ipomoea nil]
**g54791**	LOC116010267	glucose-6-phosphate 1-dehydrogenase, chloroplastic-like [Ipomoea triloba]
**g786**	LOC116020705	germin-like protein subfamily 1 member 13 [Ipomoea triloba]
**g14528**	LOC116002687	isoflavone reductase homolog isoform X3 [Ipomoea triloba]
**g48030**	LOC116025595	isovaleryl-CoA dehydrogenase, mitochondrial [Ipomoea triloba]
**g39788**	LOC116027733	glucose-6-phosphate isomerase, cytosolic [Ipomoea triloba]
**g20151**	LOC116000078	uricase isoform X1 [Ipomoea triloba]
**g49596**	LOC116025447	methylcrotonoyl-CoA carboxylase subunit alpha, mitochondrial [Ipomoea triloba]
**g797**	LOC116020930	germin-like protein subfamily 1 member 13 isoform X2 [Ipomoea triloba]
**g34609**	LOC116031766	putative lipase YDR444W [Ipomoea triloba]
**g792**	LOC109189050	PREDICTED: germin-like protein subfamily 1 member 13 [Ipomoea nil]
