# Supplementary material for: Integrated Transcriptional and Metabolomic Analysis of Factors Influencing Root Tuber Enlargement during Early Sweet Potato Development
Source: Genes (Basel). 2024 Oct 14;15(10):1319. doi: 10.3390/genes15101319 (PMC11507034; doi:10.3390/genes15101319)
Supplement: Supplementary file 1 [file genes-15-01319-s001.zip › Table S5.pdf]

Table S5. Differential accumulation metabolites shared by the four comparison groups (S1 vs S2, S3 vs S4, and S4 vs S5).

#ID	name	#ID	name
**neg_3528**	10-Hydroxycanthin-6-one	**pos_3649**	Novclobiocin 105
**neg_3824**	4-Hydroxyphenylacetaldehyde	**pos_3713**	(1xi,3xi)-1,2,3,4-Tetrahydro-1-methyl-beta-carboline-3-carboxylic acid
**neg_4319**	EMODIC ACID	**pos_3921**	Thr Phe Phe Gln
**neg_4655**	Piceid	**pos_4727**	4-(1-piperazinyl)-1H-Indole (hydrochloride)
**neg_5167**	7,8-Dihydroneopterin 2',3'-cyclic phosphate	**pos_5528**	Kaempferol 3-O-sulfate
**neg_5175**	Emodin-3-O-Sulphate	**pos_5542**	Vanillylmandelic acid
**neg_6093**	(S)-DNPA	**pos_5955**	Chlortetracycline
**neg_6097**	D-Glucosyl Indole-3-Carboxylate	**pos_6002**	Melibiitol
**neg_6100**	(-)-Glyceollin I	**pos_6022**	Homogentisate
**neg_6108**	Glucoalyssin	**pos_6023**	Monomethyl phthalate
**neg_704**	Dunalianoside B	**pos_6048**	Obtucarbamate B
**neg_7047**	Koparin	**pos_6130**	Capsoside B
**neg_7051**	Tectorigenin sodium sulfonate	**pos_7842**	Ligstroside
**pos_1165**	wilfortrine	**pos_995**	ALPHA,BETA-TREHALOSE
**pos_1372**	8-Aminooctanoic Acid		
