# Supplementary material for: Integrated Transcriptional and Metabolomic Analysis of Factors Influencing Root Tuber Enlargement during Early Sweet Potato Development
Source: Genes (Basel). 2024 Oct 14;15(10):1319. doi: 10.3390/genes15101319 (PMC11507034; doi:10.3390/genes15101319)
Supplement: Supplementary file 1 [file genes-15-01319-s001.zip › Table S6.pdf]

Table S6. Metabolome WGCNA screened for differentially accumulated metabolites.

#ID	name	#ID	name	#ID	name
**neg_10310**	Agroclavine	**neg_9603**	Morphine	**neg_3244**	Methylitaconate
**neg_10655**	Vindoline	**pos_3467**	6-Hydroxyprotopine	**neg_3388**	cis-3,4-Leucopelargonidin
**neg_10899**	Thebaine	**pos_3565**	D-Panthenol	**neg_3396**	Blue pigment
**neg_1340**	dTDP-L-megosamine	**pos_3971**	dTDP-6-deoxy-beta-L-talose	**neg_3546**	5-Deoxyleucopelargonidin
**neg_1644**	D-4'-Phosphopantothenate	**pos_5079**	3-alpha(S)-Strictosidine	**neg_3547**	Hesperetin
**neg_1651**	3-Methyl-2-oxobutanoic acid	**pos_5688**	dTDP-D-desosamine	**neg_3831**	beta-Nicotinamide D-ribonucleotide
**neg_3074**	dTDP-L-olivose	**pos_6549**	4-(3-Methylbut-2-enyl)-L-tryptophan	**neg_4293**	CMP-N-glycoloylneuraminate
**neg_3076**	dTDP-3-N,N-dimethylamino-2,3,6-trideoxy-4-keto-D-glucose	**pos_6704**	Maltol	**neg_4698**	(-)-Epicatechin
**neg_4894**	Isocorypalmine	**pos_6796**	6-O-Methyldeacetylisoipecoside	**neg_4810**	Leucocyanidin
**neg_6120**	Cathenamine	**pos_7137**	Raucaffricine	**neg_4974**	5-O-Caffeoylshikimic acid
**neg_6484**	(3R)-3-Hydroxy-2,3-dihydrotabersonine	**pos_8130**	(13S,14R)-1-Hydroxy-13-O-acetyl-N-methylcanadine	**neg_6941**	Nicotinic acid adenine dinucleotide
**neg_6741**	Pantetheine	**pos_9043**	L-Tryptophan	**pos_1133**	Niacinamide
**neg_7178**	4,21-Dehydrogeissoschizine	**neg_1238**	GDP-L-fucose	**pos_2083**	UDP-L-rhamnose
**neg_7684**	(S)-Autumnaline	**neg_1255**	GDP-mannose	**pos_3908**	CDP-abequose
**neg_7712**	Vinblastine	**neg_1331**	UDP-2-acetamido-4-dehydro-2,6-dideoxyglucose	**pos_4256**	Naringin
**neg_7809**	Laudanine	**neg_1457**	UDP-N-acetyl-alpha-D-glucosamine	**pos_4891**	Nicotinate D-ribonucleoside
**neg_7831**	Demecolcine	**neg_2944**	CMP-N-acetylneuraminate	**pos_6067**	Nicotinate D-ribonucleotide
**neg_8268**	Colcemid	**neg_2955**	Chlorogenate	**pos_6751**	4-Coumaroylshikimate
**neg_8293**	Vinorine	**neg_2995**	UDP-L-Ara4O	**pos_8008**	N-Acetylneuraminate
**neg_8938**	Allocryptopine	**neg_3130**	N,N'-Diacetylchitobiose		
